# A UV-Cured Polymer/Aluminum-Microparticle Photothermal Encapsulated Liquid-Filled Fiber Mach–Zehnder Interferometric Hot-Wire Anemometer

**DOI:** 10.3390/s26175354

**Published:** 2026-08-24

**Authors:** Cheng-Ling Lee, Wen-Hsun Hsieh, Wei-Jhou Chen, Pin Han

**Affiliations:** 1 Department of Electro-Optical Engineering, National United University, Miaoli 36003, Taiwan; m1423013@o365.nuu.edu.tw (W.-H.H.); u1223012@o365.nuu.edu.tw (W.-J.C.); 2 Graduate Institute of Precision Engineering, National Chung Hsing University, 250 Kuo Kuang Road, Taichung 40227, Taiwan; pin@dragon.nchu.edu.tw

**Keywords:** fiber-optic anemometer, hot-wire anemometry (HWA), leaky-guided fiber Mach–Zehnder interferometer (LGFMZI), polymer/aluminum-microparticle photothermal encapsulation

## Abstract

A high-sensitivity fiber-optic hot-wire anemometer based on a liquid-filled leaky-guided fiber Mach–Zehnder interferometer (LGFMZI) with UV-cured polymer/aluminum-microparticle photothermal encapsulation is proposed and experimentally demonstrated. The sensing element consists of a side-polished-fiber-assisted liquid-filled hollow-core fiber structure, in which a refractive-index-selected liquid core is introduced through a microslit to tailor the modal effective refractive-index difference and enlarge the free spectral range. The sensing region is uniformly encapsulated with a UV-cured NOA81 polymer layer containing aluminum microparticles. This encapsulation layer serves as both a photothermal conversion layer under 980 nm LD heating and a mechanical reinforcement layer. Under laser heating, the sensor is subsequently cooled by external airflow, converting wind-velocity variations into monotonic wavelength shifts. Experimental results show that, at an LD current of 80 mA corresponding to an optical power of 16 mW, the single-wavelength-dip sensor achieves a maximum airflow sensitivity of −22.922 nm/(m/s). The device also exhibits a fast transient response, with a rise time of 0.606 s and a fall time of 0.316 s. The proposed liquid-filled LGFMZI combines simple fabrication, photothermal encapsulation, high spectral readability, and stable airflow response, making it suitable for real-time fiber-optic hot-wire anemometry.

## 1. Introduction

Accurate measurement of fluid flow and wind velocity is important in many fields, including aerospace engineering, environmental monitoring, industrial process control, ventilation systems, and wind-energy applications. Conventional mechanical and electronic anemometers can be affected by electromagnetic interference, corrosion, limited installation space, and electrical-safety concerns in harsh environments. Among different flow-sensing techniques, hot-wire anemometry (HWA) is a classical and widely used method for velocity measurement because it converts flow-induced convective cooling into a measurable thermal or electrical response [1]. Optical fiber sensors provide an attractive alternative for HWA because they offer compact size, electromagnetic immunity, corrosion resistance, remote interrogation, and compatibility with miniature sensing probes. In a typical fiber-optic HWA configuration, a localized region of the fiber sensor is heated by an optical or electrical source. When airflow passes over the heated region, convective cooling changes the local temperature, and the wind velocity can be determined from the resulting optical response. Among fiber-optic anemometers, grating-based sensors are among the most widely investigated platforms because of their mature fabrication process and simple wavelength demodulation. Fiber Bragg grating (FBG)-based anemometers have been demonstrated using laser-heated FBG hot-wire sensing [2], multi-FBG curvature sensing [3], and Pitot-static-tube-assisted FBG probes [4]. To improve the hot-wire response and airflow sensitivity, modified grating structures have also been reported, including cladding-etched FBGs with silver film [5], tilted FBGs coated with carbon nanotubes or metallic layers, and high-order cladding-mode grating configurations [6,7,8]. In addition, hybrid FBG-assisted FPI flow sensors have been reported for hot-wire flow measurement and signal compensation [9], while cobalt-doped fiber-based long-period gratings have been proposed to enhance localized optical heating through 1480 nm pump absorption [10]. These grating-based approaches have shown good feasibility for fiber-optic airflow and flow sensing. However, their wavelength responses are generally limited to a relatively small range, usually from the picometer to the subnanometer scale. In many grating-based hot-wire sensors, additional treatments such as nanomaterial coating, metal-film deposition, or specialty light-absorbing fibers are primarily introduced to improve optical heating efficiency, reduce thermal mass, or enhance airflow-induced cooling, rather than increase the intrinsic temperature sensitivity of the grating itself directly. These processes may increase fabrication complexity and, in some cases, affect the mechanical robustness of the sensing element. To obtain larger optical responses, interferometric fiber sensors have been developed for hot-wire anemometry and flow sensing. Fiber Fabry–Pérot interferometers (FPIs) are particularly attractive because their compact cavities can be locally heated and used as miniature probe-type sensing heads. Various FPI-based hot-wire or thermal flow sensors have been reported, including hot-polymer FPIs [11], laser-heated Sn-microsphere air-gap FPIs [12], photothermal microcavities based on silver nanoparticles [13], optomechanical nanofilm resonators [14], silver-film-modified FPIs [15], silver-nanoparticle-solution-filled optofluidic microcavities [16], carbon-nanotube-assisted FPI microflowmeters [17], and light-source-heated FPIs [18]. These studies demonstrate that FPI and microcavity structures are effective platforms for compact fiber-optic hot-wire and flow sensing.

Compared with FBG- and FPI-based sensors, fiber Mach–Zehnder interferometers (MZIs) can provide high wavelength responsivity because their interference spectra are sensitive to small changes in the modal effective refractive-index difference. However, MZI-based flow and hot-wire anemometers have been reported much less frequently. Early work demonstrated electric-wind measurement using a Mach–Zehnder-type optical fiber interferometer, in which a small region of one interferometer arm was externally heated by an infrared CO_2_ laser and the airflow-induced cooling was detected from the interferometric response [19]. A cascaded bowknot-type tapered MZI was also used for microfluidic flow-rate sensing, but it was not operated as a hot-wire anemometer and did not include a photothermal heating layer [20]. More recently, a fiber interferometric hot-wire anemometer was demonstrated using a cobalt-doped fiber as the heated element under 1480 nm pump excitation [21]. These studies show the potential of MZI-based flow sensing. However, compact MZI hot-wire sensors with an integrated photothermal heating and packaging layer are still relatively limited. In such sensors, the heating method is closely related to the sensing structure because the sensing region must first be locally heated and then cooled by airflow. Various light-absorbing materials and heating structures have therefore been used in fiber-optic hot-wire and flow sensors, including silver or metallic films [5,15], carbon nanotube coatings [6,7,8,17,18], Sn microspheres [12], silver nanoparticles and silver nanoparticle solutions [13,16], graphene-based nanofilms or few-layer graphene [14,22], GaAs films [23], and cobalt-doped fibers [10,21]. These materials and structures can improve optical heating efficiency and strengthen the airflow-induced thermal response.

In this study, we propose and experimentally demonstrate a miniature liquid-filled leaky-guided fiber Mach–Zehnder interferometric hot-wire anemometer with a UV-cured polymer/aluminum microparticles (Al-MPs) photothermal encapsulation. Although the liquid-filled LGFMZI platform is based on our previous work and the laser-heating/airflow-cooling sensing principle has been reported previously, the primary novelty of this work lies in the proposed UV-cured polymer/aluminum-microparticle photothermal encapsulation concept and its integration with a miniature fiber-optic hot-wire sensor. The sensing element consists of a liquid-filled LGFMZI, in which a refractive-index-selected liquid is sealed inside a side-polished-fiber-assisted double-hollow-core fiber structure. The sensing region is then externally encapsulated with an NOA81 polymer layer uniformly mixed with Al-MPs. This multifunctional composite serves as a photothermal heating medium under 980 nm laser-diode irradiation and simultaneously provides protective encapsulation and mechanical reinforcement for the fragile fiber-optic sensing element. The composite is prepared by a simple mixing and UV-curing process, making it a practical approach for fabricating compact fiber-optic hot-wire sensors. The proposed photothermal encapsulation strategy is not limited to the liquid-filled LGFMZI demonstrated here and may be extended to other sealed fiber-optic sensing devices requiring localized optical heating. To the best of our knowledge, this is the first report of such an integration strategy that combines photothermal heating, protective encapsulation, and mechanical reinforcement within a single composite layer for fiber-optic hot-wire anemometry.

## 2. Device Fabrication and Sensing Principle

**Figure 1:**
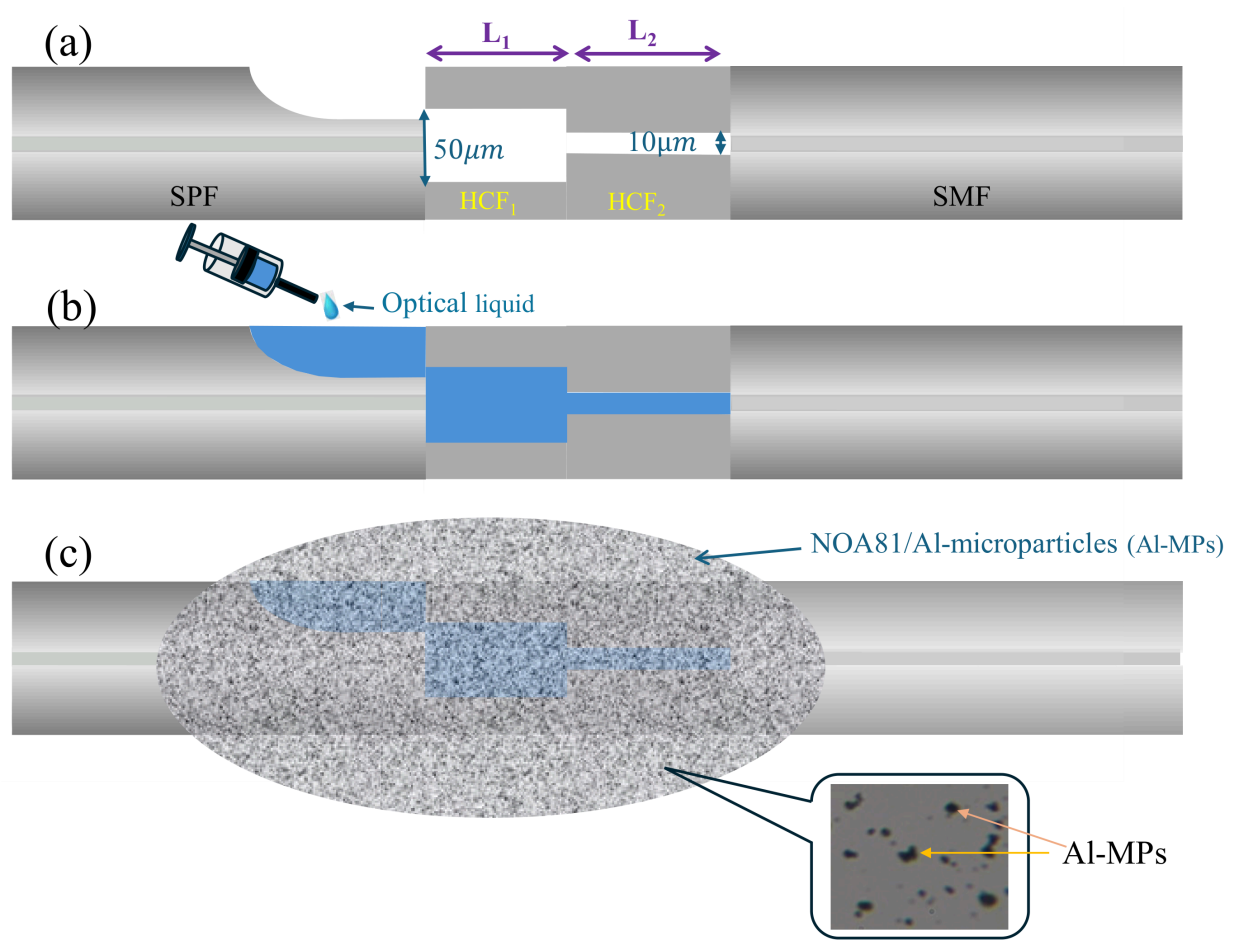
Fabrication process of the SPF-LGFMZI hot-wire anemometer: (**a**) initial air-core SPF-LGFMZI, (**b**) refractive-index-liquid-filled SPF-LGFMZI, and (**c**) polymer/Al-microparticle-encapsulated device. The inset shows a micrograph of the NOA81/Al-microparticle composite.

The proposed device is based on our previously reported liquid-filled leaky-guided fiber Mach–Zehnder interferometer (LGFMZI) configuration [24]. A schematic illustration of the sensing structure is shown in Figure 1. The device was fabricated by fusion-splicing the fiber sections in the following sequence: an input single-mode fiber (SMF), a large-core hollow-core fiber (HCF_1_; inner diameter: 50 µm, length: L_1_), a small-core hollow-core fiber (HCF_2_; inner diameter: 10 µm, length: L_2_), and an output SMF. Light launched from the input SMF first expands into HCF_1_ and then propagates through the microcore HCF_2_ section. In this cascaded HCF configuration, HCF_1_ mainly functions as a modal-field expansion region, which helps distribute the optical power between the liquid core and the silica fiber cladding. The HCF_2_ section serves as the main interference region, where the phase difference between the liquid-core leaky mode and the fiber-cladding mode is accumulated.

During fabrication, the input SMF was side polished before fusion splicing. After splicing the side-polished fiber (SPF) to HCF_1_, a microslit was naturally formed at the SPF–HCF_1_ interface, allowing the refractive-index liquid to be introduced into both HCF_1_ and HCF_2_ through capillary action and to form the liquid core. The filling opening was then hermetically sealed with a two-component epoxy resin for a closed liquid cavity. To implement hot-wire airflow sensing, the sealed sensing region was externally encapsulated with a photothermal composite layer. This layer was prepared by uniformly mixing NOA81 UV-curable polymer with aluminum (Al) microparticles with an average particle size of around 800 nm (0.8 µm) at a weight ratio of 1:0.1. The mixture was coated around the sensing region and cured under UV illumination, forming a mechanically robust polymer/Al-microparticle encapsulation with a thickness of approximately 300–400 µm and a total added weight of about 3 mg, as shown in Figure 1. Al microparticles were selected primarily for practical engineering considerations, including their ready availability, low material cost, and ease of forming a uniform composite with UV-curable NOA81, rather than because of a presumed superiority in photothermal conversion efficiency. After encapsulation, the device retained its original interference spectrum, indicating that the encapsulation process did not significantly disturb the optical interference characteristics.

The interference spectrum of the liquid-filled SPF-LGFMZI is mainly governed by the phase difference between the fiber-cladding mode and the liquid-core leaky mode. Since the refractive index of the filled refractive-index liquid is lower than that of the fused-silica fiber cladding, the liquid core operates under a leaky-guiding condition. The key relations are briefly summarized to explain the spectral design used for airflow sensing. For the two-beam interference formed in HCF_2_, the transmission dips corresponding to the destructive interference wavelength (${\lambda_{min}}^{m})$ can be expressed as [24]

(1) $${\lambda_{min}}^{m}=[\frac{2}{\left(2m+1\right)}]\cdot \left|{\Delta n}^{eff}\right|\cdot L_{2}$$

where ${\lambda_{min}}^{m}$ is the resonance wavelength of the mth transmission dip, m is the interference order, L_2_ is the effective interference length of the liquid-filled HCF_2_ sensing region, and ${\Delta n}^{eff}$ denotes the effective refractive-index difference between the fiber-cladding mode and the liquid-core leaky mode. The free spectral range (FSR) between two adjacent dips can be approximated as

(2) $$FSR=\frac{\left(\lambda_{1}\cdot \lambda_{2}\right)}{\left(\left|{\Delta n}^{eff}\right|L_{2}\right)}$$

where λ_1_ and λ_2_ are the resonance wavelengths of two adjacent transmission dips. According to this relation, the FSR can be enlarged by reducing |${\Delta n}^{eff}$| or shortening L_2_. In this work, two spectral conditions were designed for comparison. Sensor 1 was designed to exhibit a multiple-wavelength-dip spectrum, whereas Sensor 2 was designed to produce a single-wavelength-dip spectrum by selecting a suitable refractive-index liquid and HCF_2_ length (L_2_). For the single-wavelength-dip device, the FSR is expanded beyond the 1250–1650 nm interrogation window, leaving only one clear resonance dip for wavelength tracking. This simplified spectrum is especially useful for hot-wire airflow sensing because laser heating and airflow cooling can induce large wavelength shifts, which may otherwise cause tracking ambiguity in dense multi-dip spectra.

**Figure 2:**
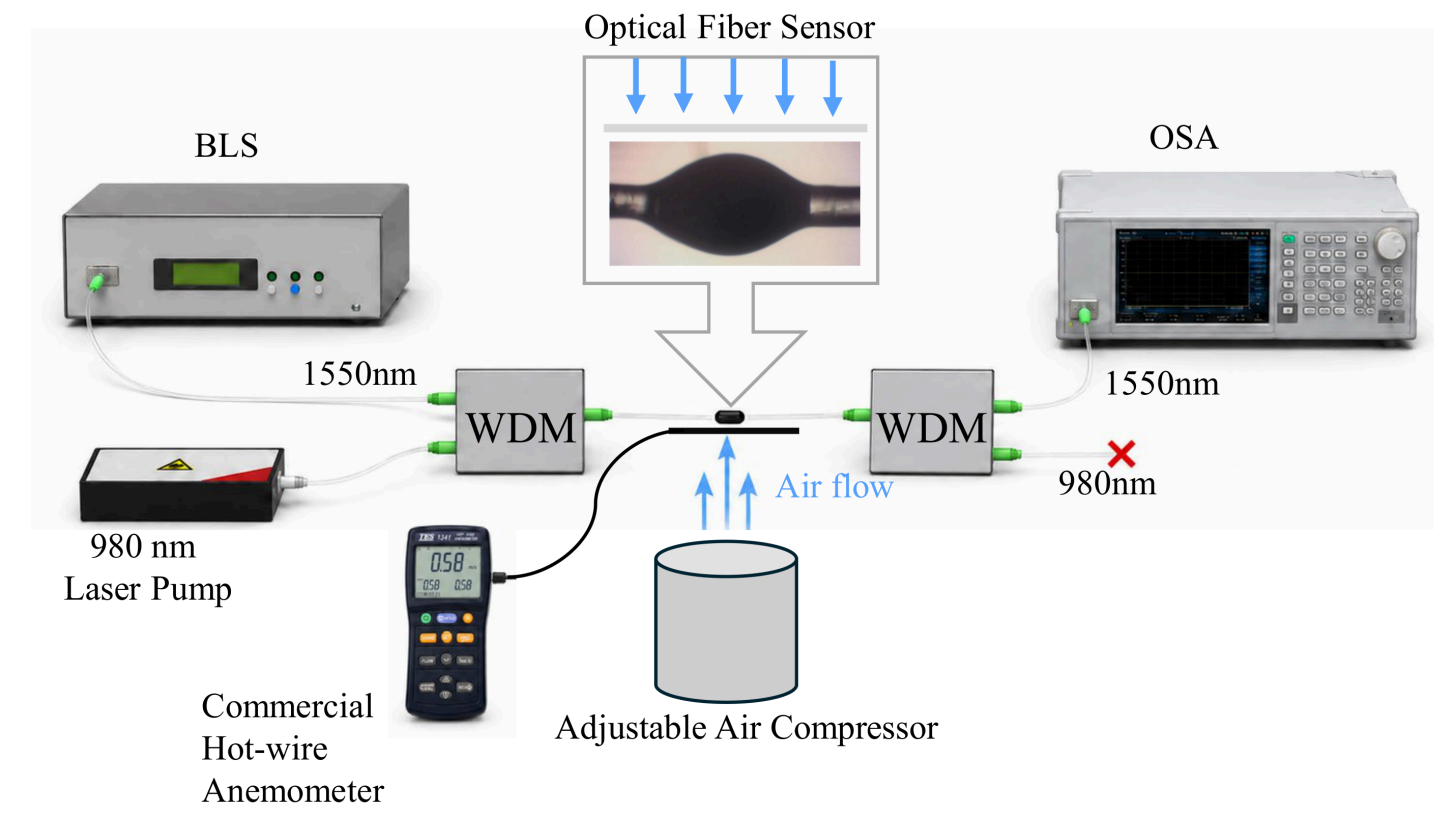
Experimental setup for airflow-speed measurement using the polymer/Al-microparticle-encapsulated LGFMZI. The 980 nm pump laser heats the sensing head, and the BLS/OSA monitors the transmission spectrum. A commercial hot-wire anemometer is used as a reference.

Figure 2 shows the experimental setup used for airflow-speed measurements. The measurement system consists of a broadband light source (BLS), a 980 nm pump laser diode (LD), two 980/1550 nm wavelength-division multiplexers (WDMs), and an optical spectrum analyzer (OSA, Anritsu-MS9740A). The BLS was launched into the fiber device to monitor the transmission interference spectrum, while the 980 nm pump light was introduced through the WDM to optically heat the sensing region for hot-wire anemometry. In the study, the polymer/Al-microparticle encapsulation is applied to the liquid-filled LGFMZI as an external photothermal and protective layer. The dispersed Al-MPs are expected to enhance the interaction with the 980 nm pump light and generate localized photothermal heating, while the UV-cured polymer fixes microparticles around the fiber and mechanically reinforces the sealed sensing element. Therefore, the encapsulation layer not only converts part of the coupled pump energy into heat but also helps stabilize the liquid-filled fiber structure during airflow exposure. Under 980 nm laser heating, a localized thermal field is established around the sensing region, raising the temperature (T) of the fiber structure and the refractive-index liquid inside the hollow-core region. As the T increases, the refractive index of the refractive-index liquid decreases because of its negative thermo-optic coefficient (TOC). In contrast, the refractive index of the fused-silica fiber cladding increases slightly owing to its positive TOC. This opposite thermo-optic response changes the modal effective refractive-index difference, |${\Delta n}^{eff}$|, thereby redshifting the interference spectrum. During wind-speed characterization, the LGFMZI sensing element was placed in a perpendicular crossflow configuration. The external airflow removes heat from the encapsulated sensing region through convective cooling, decreasing the local T and inducing a spectral blueshift. The combined optical signal then passes through the output WDM; the 1550 nm signal is routed to the OSA for spectral monitoring, while the residual 980 nm pump light is separated from the detection path to protect the instrument. A commercial hot-wire anemometer was positioned near the sensing head to record the real-time reference wind speed.

## 3. Experimental Results and Discussion

**Figure 3:**
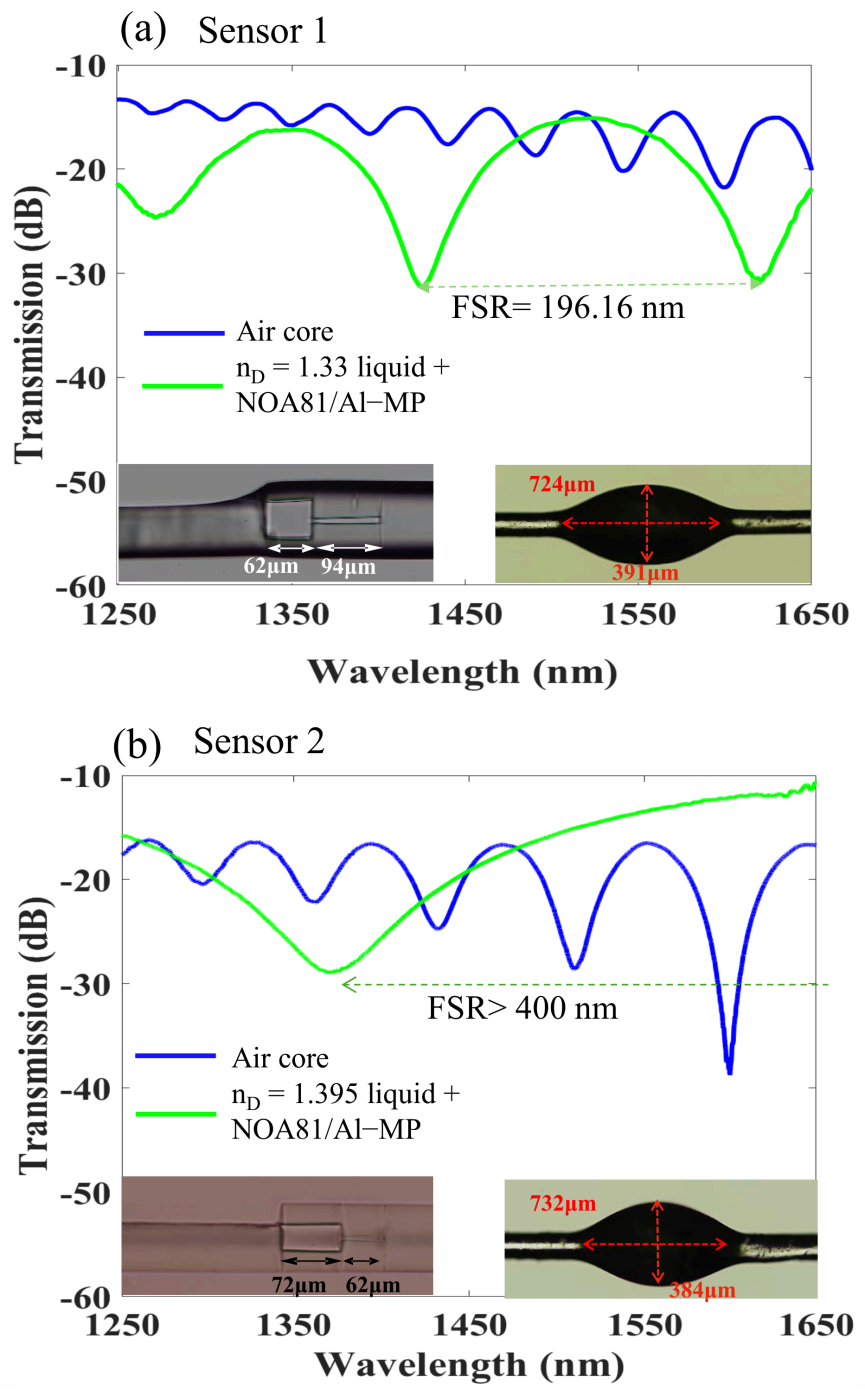
Initial transmission spectra and microscopic images of the proposed sensors: (**a**) Sensor 1 with a multiple-wavelength-dip spectrum using the n_D_ = 1.33 refractive-index liquid and (**b**) Sensor 2 with a single-wavelength-dip spectrum using the n_D_ = 1.395 refractive-index liquid. Blue and green curves represent the air-core and liquid-filled/encapsulated states, respectively.

Before evaluating the airflow response, the initial spectral characteristics and physical dimensions of the fabricated sensing elements were first examined. Two liquid-filled SPF-LGFMZI devices were prepared with different refractive-index liquids and HCF lengths to obtain different interference conditions. Sensor 1 was designed to exhibit a multiple-wavelength-dip spectrum, whereas Sensor 2 was designed to provide a single-wavelength-dip spectrum within the 1250–1650 nm interrogation window. Both devices were then externally encapsulated with the polymer/Al-microparticle encapsulation to form the completed hot-wire sensing heads. Figure 3 shows the microscopic images and initial transmission spectra of the two sensors under zero-airflow conditions. As shown in Figure 3a, Sensor 1 consists of a large-core HCF section with L**_1_** = 62 µm and an inner diameter of 50 µm, followed by a small-core HCF section with L**_2_** = 94 µm and an inner diameter of 10 µm. After filling with the refractive-index liquid of n**_D_** = 1.33, Sensor 1 exhibits a multiple-dip interference spectrum with an FSR of 196.16 nm across the 1250–1650 nm wavelength range. This spectral behavior results from the relatively large $\left|{∆n}^{eff}\right|L_{2}$.

In contrast, Sensor 2 was fabricated with L**_1_** = 72 µm and L**_2_** = 62 µm, as shown in Figure 3b. When a refractive-index liquid of n**_D_** = 1.395 was introduced, the effective refractive-index difference was reduced, and the FSR could be enlarged to beyond 400 nm. As a result, only one prominent interference dip remained within the interrogation window, forming a clear single-wavelength dip spectrum suitable for wavelength tracking. The insets in Figure 3 also show the completed polymer/Al-microparticle encapsulation around the sensing heads. The polymer/Al-microparticle encapsulated regions of Sensor 1 and Sensor 2 exhibited cross-sectional dimensions of approximately 724 µm × 391 µm and 732 µm × 384 µm, respectively. The added encapsulation weight was approximately 0.003 g, corresponding to 3 mg. These comparable encapsulation dimensions and weights indicate that both devices were packaged under similar photothermal loading conditions for the subsequent laser-heating and airflow-cooling measurements.

**Figure 4:**
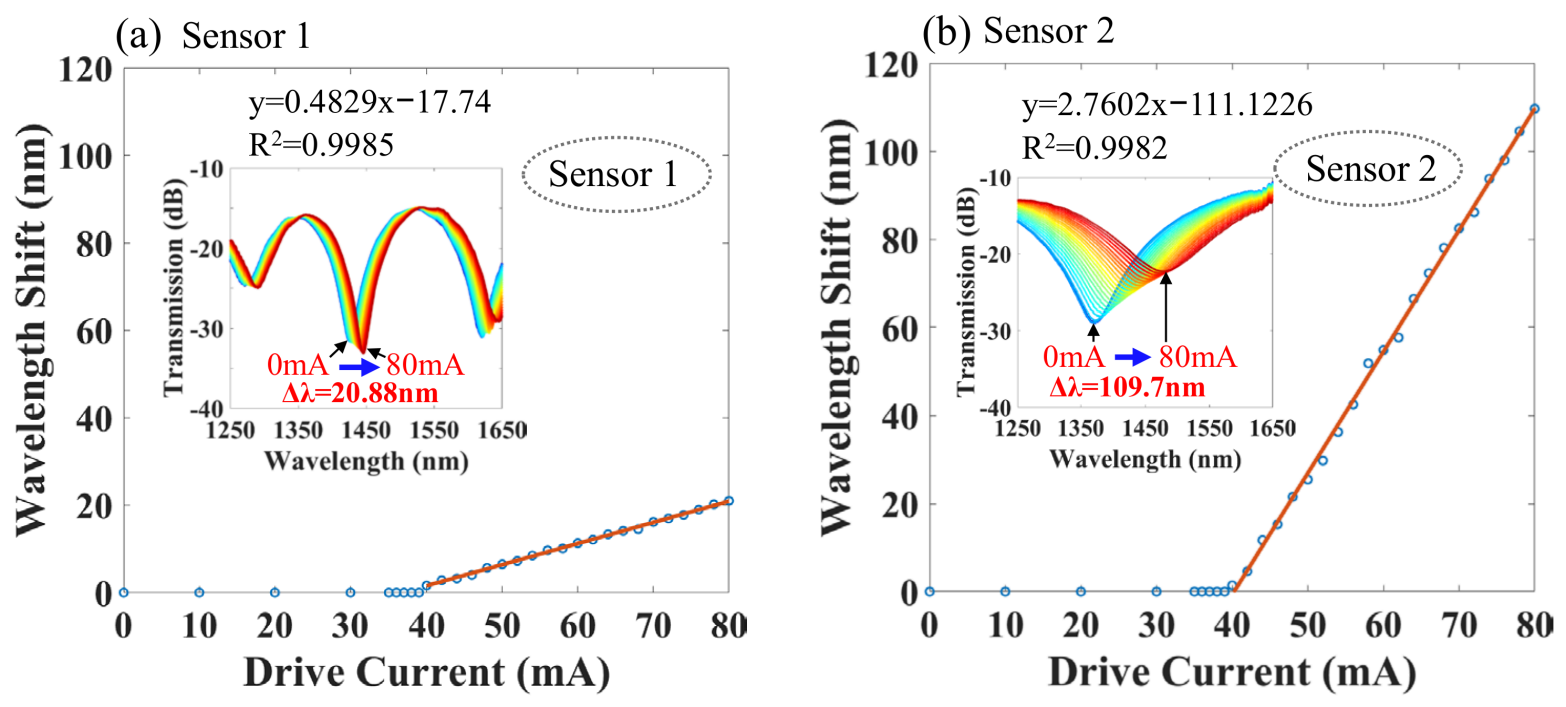
Laser-heating responses of Sensor 1 and Sensor 2 under 980 nm pump currents from 0 to 80 mA. Insets show the corresponding spectral redshifts.

The laser-heating characteristics of the two polymer/Al-microparticle-encapsulated sensing elements were first examined before airflow measurements. Figure 4 shows the wavelength shifts of Sensor 1 and Sensor 2 under different 980 nm pump laser driving currents from I = 0 to 80 mA at an ambient T of 25 °C and zero airflow. When the 980 nm pump light is coupled into the sensing region, the polymer/Al-MPs encapsulation acts as the photothermal conversion layer. The dispersed Al-MPs enhance the interaction with the pump light and generate localized heating, while the UV-cured polymer helps distribute the heat around the encapsulated fiber structure. As a result, the hollow-core region and the refractive-index liquid are heated. As the T rises, the refractive index of the liquid core decreases because of the negative TOC of the refractive-index liquid. This modifies the modal effective refractive-index difference between the liquid-core leaky mode and the fiber-cladding mode, thereby redshifting the interference spectrum. For Sensor 1 with the multiple-wavelength-dip spectrum, as shown in Figure 4a, the resonance dips shift toward longer wavelengths as the laser current increases. A total wavelength shift of 20.88 nm is obtained at a driving current of 80 mA. However, because several dips appear within the interrogation window, it is not always clear which dip should be selected and continuously tracked during the spectral redshift.

In contrast, Sensor 2 exhibits a much larger laser-heating-induced wavelength shift while maintaining a clear single-wavelength-dip performance, as shown in Figure 4b. At the same driving current of I = 80 mA, the single-wavelength-dip spectrum shows a total redshift of 109.7 nm. Since the two sensors have comparable polymer/Al-microparticle encapsulation dimensions and similar added encapsulation weights, they are expected to receive similar photothermal heating under the same 980 nm pump condition. Sensor 2 (single-wavelength-dip) was designed to exhibit a higher wavelength responsivity to temperature than Sensor 1 (multiple-wavelength-dip); it produced a larger airflow-induced wavelength shift under comparable photothermal heating conditions. In particular, the refractive-index liquid used in Sensor 2 has a slightly larger negative TOC, with dn**_D_**/dT ≈ −3.45 × 10^−4^ °C^−1^, compared with dn**_D_**/dT ≈ −3.37 × 10^−4^ °C^−1^ for the liquid used in Sensor 1. This larger negative TOC can contribute to a stronger T-induced refractive-index variation in the liquid core. However, the TOC difference alone is not sufficient to account for the much larger wavelength shift of Sensor 2. The shorter HCF**_2_** length also changes the interference order and resonance condition described by Equation (1), allowing the T-induced refractive-index variation to be converted into a larger resonance-wavelength shift. Meanwhile, the enlarged FSR of Sensor 2 keeps the shifted spectrum in a single-wavelength-dip form, preventing adjacent-dip overlaps during the large thermal redshift.

**Figure 5:**
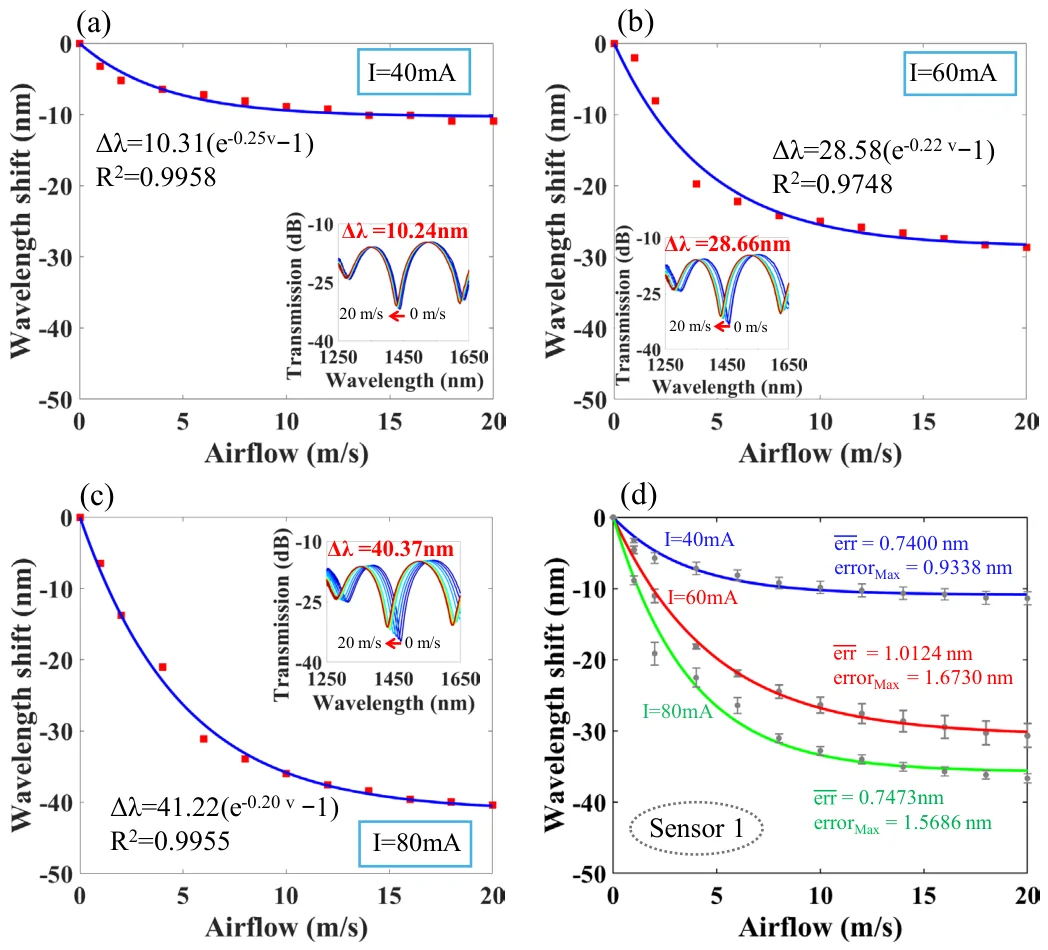
Airflow-cooling responses of Sensor 1 with the multiple-wavelength-dip spectrum under 980 nm laser driving currents of (**a**) 40 mA, (**b**) 60 mA, and (**c**) 80 mA. The insets show the corresponding spectral blueshifts after airflow cooling reaches a steady state. (**d**) Wavelength shifts obtained from five repeated measurements over an airflow-speed range of 0–20 m/s with standard deviation error bars.

Figure 5 shows the airflow-cooling responses of Sensor 1 with the multiple-wavelength-dip spectrum under fixed 980 nm laser driving currents of I = 40, 60, and 80 mA, corresponding to output laser powers of 1 mW, 11.1 mW, and 16 mW, respectively. All airflow experiments were performed in an air-conditioned laboratory maintained at approximately 25 °C with a relative humidity of approximately 50% RH to provide stable environmental conditions during the measurements. Before airflow was applied, the sensing region was heated by the 980 nm pump laser through the polymer/Al-microparticle encapsulation. When external airflow passed across the sensing region, convective cooling removed heat from the encapsulated fiber structure and decreased the local T of the hollow-core region. Because the refractive-index liquid has a negative TOC, the decrease in T increases the refractive index of the liquid core. The change modifies the modal effective refractive-index difference between the liquid-core leaky mode and the fiber-cladding mode, causing the interference dips to shift toward shorter wavelengths.

As shown in Figure 5a–c, the multiple-wavelength-dip spectrum exhibits clear blue shifts as the airflow speed increases from 0 to 20 m/s. After the airflow-cooling process reached a steady thermal equilibrium, total blueshifts of 10.24, 28.66, and 40.37 nm were obtained under driving currents of 40, 60, and 80 mA, respectively (as shown in the insets). The larger wavelength shift at higher driving current results from stronger 980 nm laser heating, which makes the sensing region more responsive to airflow cooling. To evaluate the repeatability of the airflow response, five repeated measurements were performed under the same flow conditions, and the corresponding wavelength shifts with standard deviation error bars are summarized in Figure 5d. The average errors at I = 40, 60, and 80 mA cases were 0.7400, 1.0124, and 0.7473 nm, respectively, while the maximum errors were 0.9338, 1.6730, and 1.5686 nm, respectively. These results indicate that Sensor 1 has acceptable repeatability under repeated airflow measurements. However, because several interference dips coexist within the 1250–1650 nm interrogation window, the simultaneous blueshift of multiple dips can make wavelength tracking less straightforward, especially when the spectral displacement becomes large. This observation motivates the use of a single-wavelength-dip spectrum to simplify demodulation in the following measurements.

**Figure 6:**
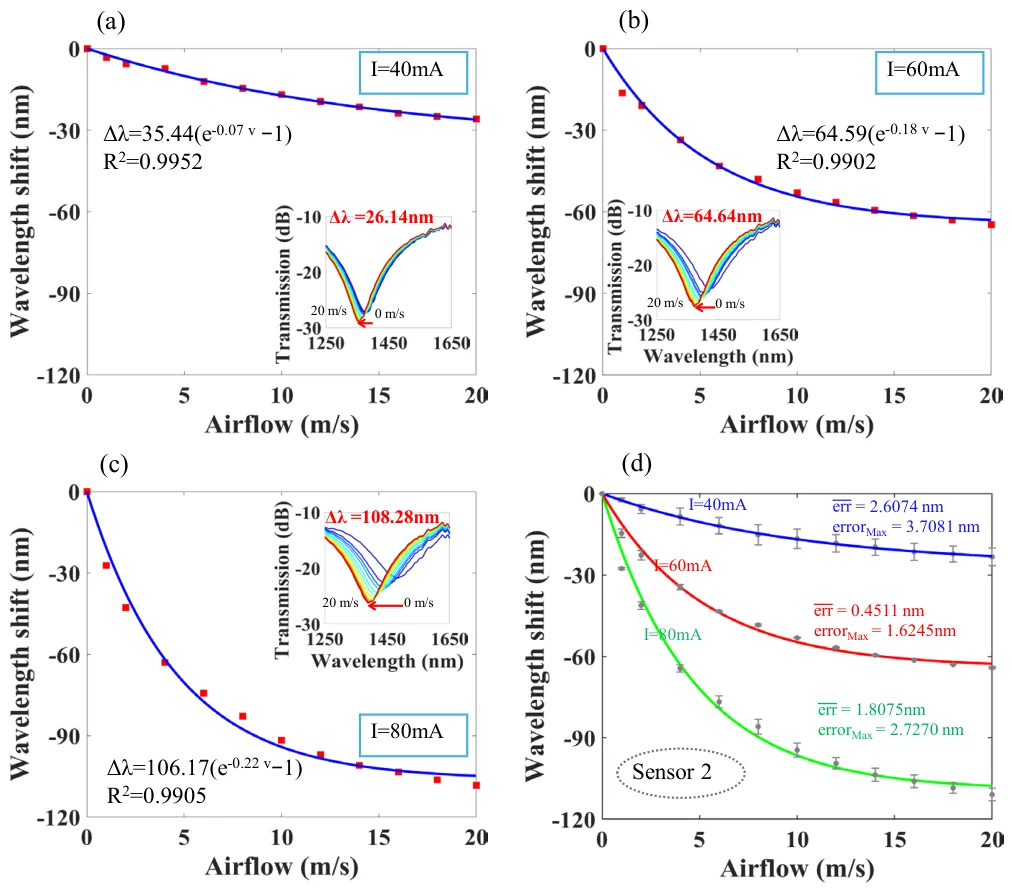
Airflow-cooling responses of Sensor 2 with the single-wavelength-dip spectrum under 980 nm laser driving currents of (**a**) 40 mA, (**b**) 60 mA, and (**c**) 80 mA. The insets show the corresponding spectral blueshifts after airflow cooling reaches a steady state. (**d**) Wavelength shifts obtained from five repeated measurements over an airflow-speed range of 0–20 m/s with standard deviation error bars.

Figure 6 shows the airflow-cooling responses of Sensor 2 with the single-wavelength-dip spectrum under the same 980 nm laser driving currents of I = 40, 60, and 80 mA. As the airflow speed increases from 0 to 20 m/s, the single wavelength dip shifts continuously toward shorter wavelengths. The total blueshifts are 26.14, 64.64, and 108.28 nm at driving currents of I = 40, 60, and 80 mA, respectively, which are much larger than those of Sensor 1 under the same heating conditions. This larger response is mainly related to the stronger thermal modulation of the interference condition in Sensor 2, including the slightly larger negative TOC of the refractive-index liquid and the resonance condition determined by the shorter HCF_2_ length. In addition, the isolated single-wavelength-dip profile makes the shifted dip easier to identify and track during airflow cooling. The repeatability of Sensor 2 was also evaluated from five repeated measurements, as shown in Figure 6d. The average errors at I = 40, 60, and 80 mA are 2.6074, 0.4511, and 1.8075 nm, respectively, and the corresponding maximum errors are 3.7081, 1.6245, and 2.7270 nm. The relatively larger error at 40 mA is mainly attributed to the low heating power. Under this condition, the temperature difference between the sensing region and the ambient environment is small, so the wavelength shift is more easily affected by environmental fluctuations. Even with this larger error at low pump power, Sensor 2 still shows a much larger airflow-induced wavelength shift than Sensor 1 under the same heating conditions.

**Figure 7:**
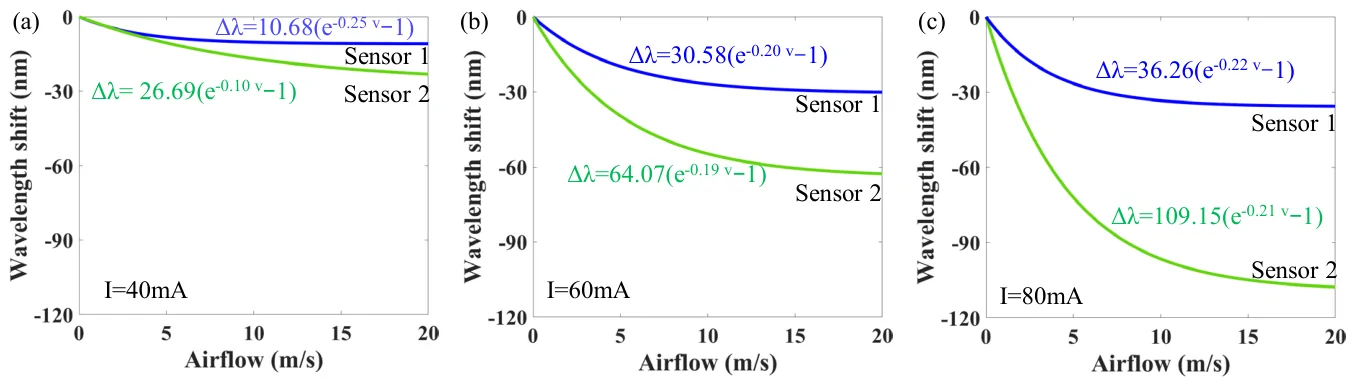
Comparison of the fitted airflow-cooling responses of Sensor 1 and Sensor 2 under 980 nm laser driving currents of (**a**) 40 mA, (**b**) 60 mA, and (**c**) 80 mA. Each fitted curve was obtained from the average wavelength shifts of five repeated measurements.

Figure 7 compares the airflow-cooling responses of Sensor 1 and Sensor 2 under the same 980 nm laser driving currents. The fitted curves were obtained from the average wavelength shifts of five repeated measurements, and the corresponding fitting equations are shown in the figure. Under all tested driving currents, Sensor 2 exhibits a larger airflow-induced wavelength shift than Sensor 1. This comparison shows that, under the same heating and airflow conditions, the single-wavelength-dip Sensor 2 provides a more sensitive airflow response than the multiple-wavelength-dip Sensor 1.

To further compare the airflow responses of the two sensors, the sensitivity was calculated from the first derivative of the fitted wavelength-shift curve. The wavelength-shift sensitivity, S, is defined as

(3) $$S=\frac{d(\Delta \lambda )}{dv}$$

where Δλ is the airflow-induced wavelength shift, and *v* is the airflow speed. Since the wavelength shift exhibits nonlinear saturation behavior as the airflow speed increases, the sensitivity is not a constant value but varies with *v*.

**Figure 8:**
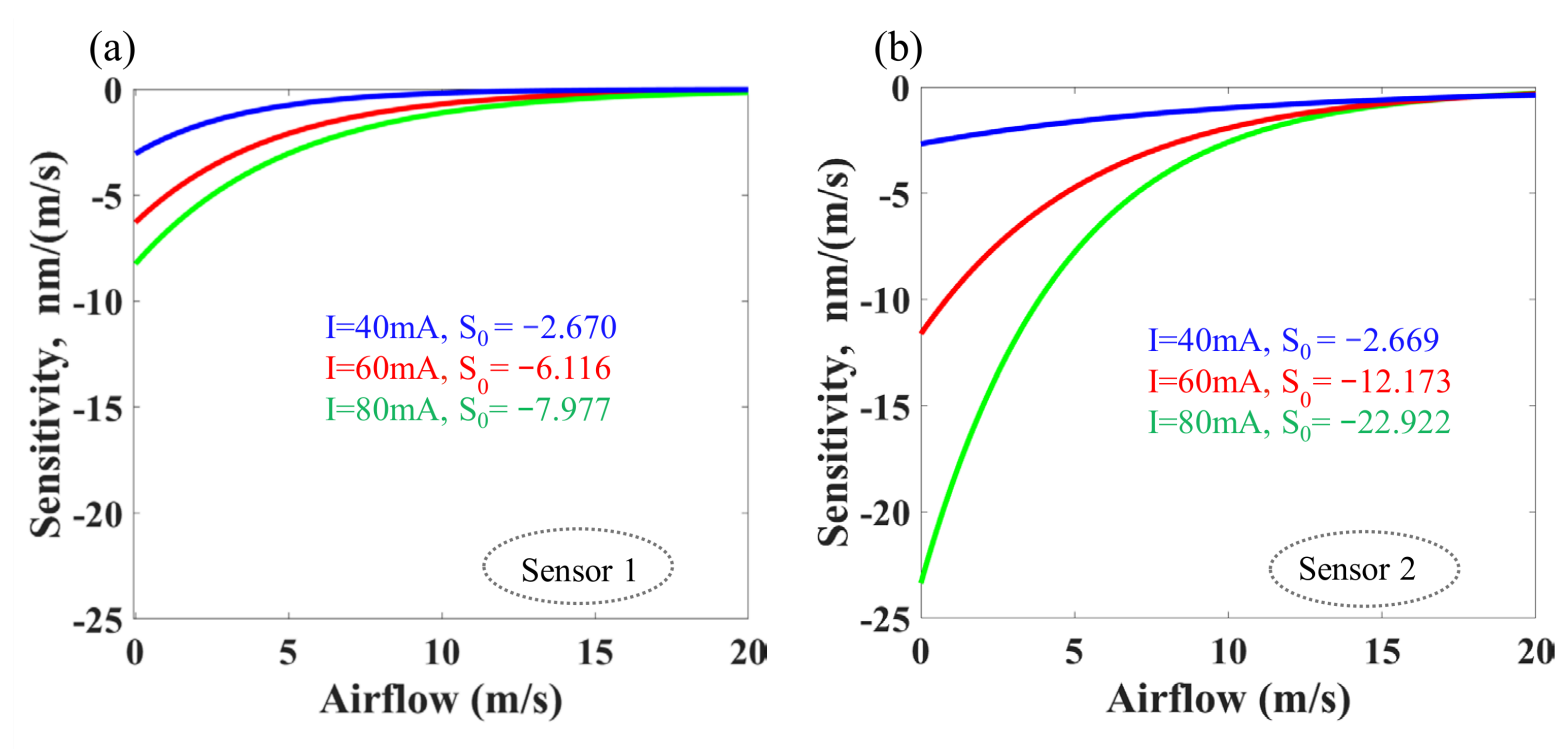
Sensitivity curves calculated from the fitted average wavelength-shift responses of five repeated measurements for (**a**) Sensor 1 with the multiple-wavelength-dip spectrum and (**b**) Sensor 2 with the single-wavelength-dip spectrum under different 980 nm laser driving currents.

Figure 8 shows the sensitivity curves of Sensor 1 and Sensor 2 under different 980 nm laser driving currents. The curves were obtained by fitting the average wavelength shifts from five repeated measurements and then differentiating the fitted curves. For both sensors, the absolute sensitivity is highest at low airflow speed and gradually decreases as the airflow speed increases. This is because the cooling effect is stronger at low airflow speed, while the wavelength shift gradually becomes saturated at higher airflow speed. For Sensor 1 with the multiple-wavelength-dip spectrum, the initial sensitivities near zero airflow speed (S_0_) are −2.670, −6.116, and −7.977 nm/(m/s) at driving currents of I = 40, 60, and 80 mA, respectively. For Sensor 2 with the single-wavelength-dip spectrum, the corresponding (S_0_) values are −2.669, −12.173, and −22.922 nm/(m/s), respectively. At I = 40 mA, the two sensors show nearly the same initial sensitivity. However, at I = 60 and 80 mA, Sensor 2 gives a much larger response. In particular, at 80 mA, the initial sensitivity of Sensor 2 is about 2.9 times that of Sensor 1. These results show that, under sufficient laser heating, Sensor 2 provides a stronger airflow-induced wavelength response while keeping a clear single-wavelength-dip spectrum for wavelength tracking.

**Figure 9:**
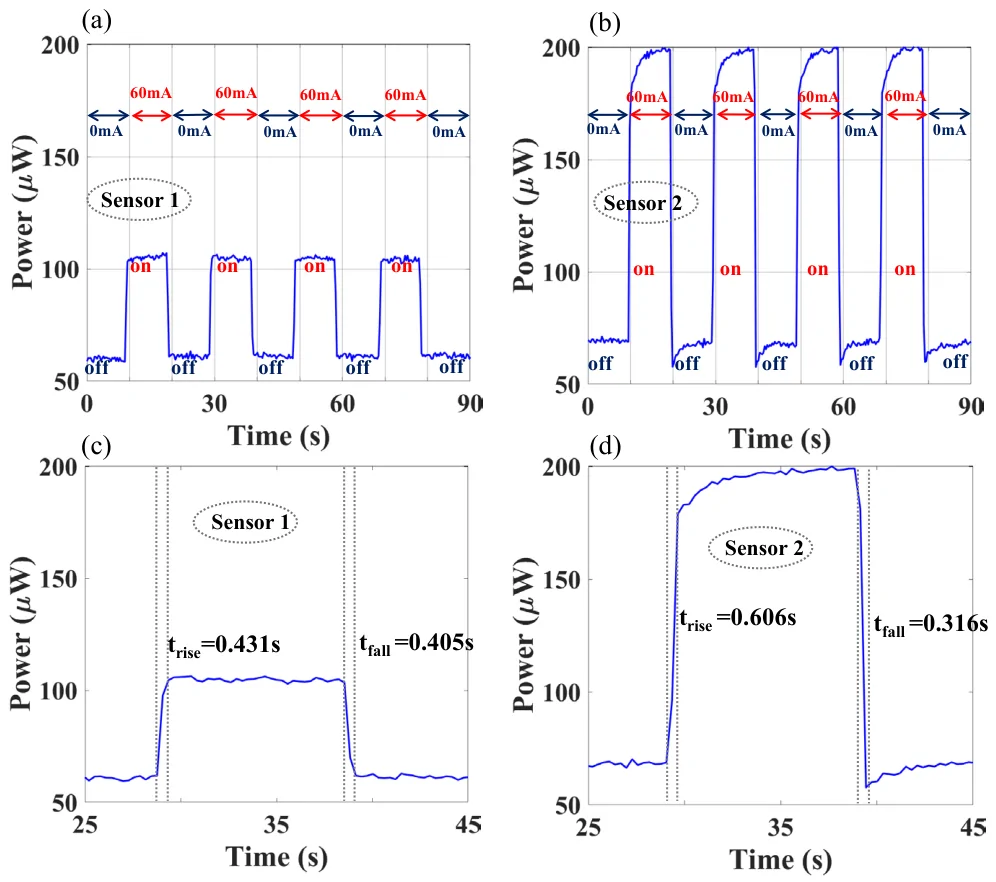
Transient optical-power responses of the polymer/Al-microparticle-encapsulated SPF-LGFMZI sensors under repeated 980 nm pump-laser switching between 0 and 60 mA. (**a**) Repeated switching responses of Sensor 1. (**b**) Repeated switching responses of Sensor 2. (**c**) Enlarged transient response of Sensor 1 used to estimate the rise and fall times. (**d**) Enlarged transient response of Sensor 2 used to estimate the rise and fall times. The rise and fall times were determined using the 10–90% and 90–10% criteria, respectively.

Figure 9 shows the transient responses of the two polymer/Al-microparticle-encapsulated SPF-LGFMZI sensors when the 980 nm pump laser was repeatedly switched between 0 and 60 mA. As shown in Figure 9a,b, both sensors exhibit repeatable optical-power changes during the on/off cycles, indicating that the polymer/Al-microparticle encapsulation can provide stable laser-induced heating. The enlarged responses in Figure 9c,d were used to estimate the rise and fall times. For Sensor 1 with the multiple-wavelength-dip spectrum, the rise and fall times are 0.431 s and 0.405 s, respectively. For Sensor 2 with the single-wavelength-dip spectrum, the corresponding values are 0.606 s and 0.316 s. These subsecond response times show that the encapsulated sensing heads can be heated and cooled rapidly under pump-laser switching. Although Sensor 2 exhibits a slightly longer rise time, its shorter fall time indicates faster cooling after the pump laser is turned off.

These results demonstrate that the uniformly mixed NOA81 polymer and Al-MPs form an effective photothermal encapsulation layer. The encapsulation not only reinforces the sensing element mechanically but also enables rapid thermal exchange during laser heating and cooling. Combined with the large wavelength response and simplified single-wavelength-dip demodulation discussed above, the proposed sensor configuration is promising for real-time hot-wire airflow monitoring. It is worth mentioning that the photothermal effect is evaluated indirectly through the laser-heating-induced wavelength shifts and the repeatable transient responses. Direct measurements of optical absorption efficiency, photothermal conversion efficiency, temperature rise, and comparisons with pure NOA81 polymer are beyond the scope of the present study and will be investigated in future work.

To better position the proposed sensor with respect to recently reported photothermal fiber-optic hot-wire anemometers, a comparison is summarized in Table 1. As shown in Table 1, the proposed sensor combines high wavelength sensitivity, fast transient response, simple fabrication, and a multifunctional photothermal encapsulation strategy within a compact fiber-optic platform.

## 4. Conclusions

We have proposed and experimentally demonstrated a liquid-filled leaky-guided fiber Mach–Zehnder interferometric (LGFMZI) hot-wire anemometer with a UV-cured polymer/Al-microparticle photothermal encapsulation. To the best of our knowledge, this is the first demonstration of using a UV-cured polymer uniformly mixed with Al-MPs as both the photothermal heating layer and the external protective encapsulation for a liquid-filled LGFMZI hot-wire anemometer. The NOA81 polymer/Al-microparticle composite was prepared by simple mixing, coating, and UV curing. Under 980 nm laser-diode irradiation, this encapsulation layer provides localized photothermal heating while fixing and protecting the miniature liquid-filled sensing structure. Two sensing elements were compared under the same laser-heating and airflow-cooling conditions. At a driving current of 80 mA, the multiple-wavelength-dip sensor showed a total blueshift of 40.37 nm, whereas the single-wavelength-dip sensor achieved a much larger blueshift of 108.28 nm. The corresponding maximum initial sensitivities were −7.977 and −22.922 nm/(m/s) for Sensor 1 and 2, respectively. For Sensor 2, the single-wavelength-dip spectrum also provides a clearer feature for wavelength tracking and reduces the ambiguity caused by adjacent dips. The transient test further confirmed a fast response, with rise and fall times of 0.606 s and 0.316 s. These results show that the proposed configuration offers high airflow sensitivity, simple fabrication, protective photothermal encapsulation, and clear wavelength demodulation for real-time fiber-optic hot-wire airflow monitoring. Future work will focus on systematic evaluations of directional airflow response, extended airflow ranges, and long-term environmental reliability to further validate the proposed photothermal encapsulation strategy for practical applications.

## Figures and Tables

**Table 1:** Comparison of representative photothermal fiber-optic anemometers.

Fiber Device	Sensing Mechanism	Fabrication Complexity	Sensitivity	Response Time
Liquid-filled LGFMZI+ NOA81/Al-microparticle (this work)	The NOA81/Al-microparticle layer provides photothermal heating, while airflow cooling changes the liquid-core refractive index and resonance wavelength.	Moderate	Maximum initial sensitivity: −22.922 nm/(m/s)	Pump-switching transient: rise 0.606 s; fall 0.316 s
Silver-coated cladding-etched FBG [5]	The silver film absorbs the 1480 nm laser for photothermal heating, while airflow cooling shifts the FBG Bragg wavelength.	Complex	Maximum sensitivity −3.180 nm/(m/s) at 0.1 m/s and laser power of 200 mW	N/R
CNT-coated highly tilted FBG-SPR [6]	CNT absorbs pump light for photothermal heating, while airflow cooling changes the SPR wavelength and intensity.	Complex	Average sensitivity: −1.3815 nm/(m/s); average loss sensitivity: −9.0769 dB/(m/s).	~3 s
SWCNT-coated tilted FBG [7]	SWCNT photothermal heating and airflow cooling modulate the transmitted optical intensity.	Complex	7.425 dBm/(m/s), intensity-based sensitivity	~5.0 s
Sn-microsphere air gap FPI [12]	A laser-heated Sn microsphere is cooled by airflow, changing the air-gap interference condition.	Moderate	0.62 nm/(m/s) (average over 0–10 m/s at 45 mW)	N/R
Silver-coated polymer air gap FPI [15]	The silver film absorbs the 980 nm laser to heat the polymer FPI, while airflow cooling shifts the interference wavelength.	Moderate	Average sensitivity of 3.302 nm/(m/s) over 0–20 m/s using a 270 nm Ag film	Pump-switching transient: rise time 0.14 s; fall time 0.98 s
Light-source-heated polymer FPI [18]	Light-source heating and airflow cooling produce a wavelength shift in the polymer FPI.	Moderate	Up to −3.13 nm/(m/s) at low speeds; ~−0.2 nm/(m/s) at higher speeds (0–7 m/s)	Response: 0.25 s; Recovery: 0.58 s
Cobalt-doped-fiber MZI [21]	The cobalt-doped fiber absorbs pump light, and airflow cooling changes the phase difference between the MZI arms.	Simple	−5.13 nm/(m/s) at 0.1 m/s; −2.44 nm/(m/s) at 0.5 m/s	N/R

## Data Availability

The data presented in this study are available on request from the corresponding author.
